# Stereotactic body radiotherapy for central lung tumors, yes we can!

**DOI:** 10.1186/s13014-018-1017-y

**Published:** 2018-04-25

**Authors:** Yasmin Korzets ceder, Eyal Fenig, Aron Popvtzer, Nir Peled, Mordechai R. Kramer, Milton Saute, Dima Bragilovsky, Tzippy Schochat, Aaron M. Allen

**Affiliations:** 1 Institute of Oncology, Davidoff Center, Rabin Medical Center, Petach Tikva, Israel; 2 Sackler Faculty of Medicine, Tel-Aviv University, Tel Aviv, Israel; 3 Division of pulmonology, Rabin Medical Center, Petach Tikva, Israel; 4 Department of Cardiothoracic Surgery, Rabin Medical Center, Petach Tikva, Israel; 5 Research and statistics unit, Rabin Medical Center, Petach Tikva, Israel

## Abstract

**Background:**

SBRT is standard therapy for early stage lung cancer. Toxicity in central tumors has been a concern. RTOG 0813 showed that central SBRT is safe and effective. We report our experience with central SBRT.

**Methods:**

We reviewed the records of patients treated with SBRT for central lung tumors (< 2 cm of the carina). Patients included primary lung cancer and recurrence following surgery and\ or conventional radiotherapy. All patients underwent 4DCT simulation and treatment planning was done with IMRT or VMAT techniques. Dose to the PTV was prescribed to the 95% isodose line.

**Results:**

Seventy patients, between 5/09 and 4/13, were treated. Patients had early non-small cell lung cancer (*n* = 13) or locally recurrent lung cancer (*n* = 29) and pulmonary oligometastases (*n* = 28). Fifty-seven percent of the patients received BED of 132 with a schedule of 60Gy in 12 Gy fractions. Median follow up time was 18.3 months, 4/70 patients experienced local failure (6%). Median OS for the whole cohort was 4.6 years (CI 3-7 years). Ten patients had grade 1-2 radiation pneumonitis. One patient developed fatal bronchial bleeding.

**Conclusions:**

SBRT for central tumors is safe and effective in patients with central disease, reiradiation, recurrence following surgery and in oligometastes.

## Background

SBRT (Stereotactic Body Radiation Therapy) is a well-established treatment for early stage medically inoperable non small cell lung carcinoma (NSCLC). SBRT has proven its place with excellent local control and limited toxicity [1–3].

In peripheral lung tumors, SBRT is the primary treatment option for medically inoperable stage I NSCLC. Treatingumors with central location (within 2 cm of the carina) have been more challenging. Initially, concerns were raised in treating central tumors due to toxicity, specifically bleeding and necrosis as described by Timmerman et al. [1]. Patients with centrally located tumors were excluded from the landmark RTOG 0236 trial.

More recently data from single institutions has emerged showing central SBRT to be safe and effective, if the fractionation schedule is modified. Recently, the RTOG 0813 has been reported and shown that in the cooperative groups setting central SBRT is safe and effective.

The optimal fractionation regimen in treating these central tumors remains unclear and has led to various regimens used worldwide based on institutional experience. Published reports have shown various regimes using BED of 80-132Gy. In light of this uncertainty we have herein reported our own large experience in SBRT for central tumors.

## Methods

Between February 2009 to April 2013, the complete radiotherapy records of 70 consecutive centrally located tumors were reviewed with IRB approval. Data collection was done based on a retrospective chart review. Toxicity was determined based on the common terminology criteria for Adverse Events version 4.0 (CTCAE 4.0).

Central tumors were defined as all tumors within 2 cm of the proximal bronchial tree or within 2 cm from major vessels (aorta, upper mediastinal vessels, and pulmonary artery extending to the tertiary bronchus), esophagus, heart, trachea, pericardium, brachial plexus, or vertebral body. Ultracentral tumors were defined as tumors with direct contact with the primary bronchial tree or espohagus.

Patients were offered SBRT for a variety of reasons, including high surgical risk (cardiac disease, poor pretreatment pulmonary function, and inadequate predicted postoperative pulmonary function) and - patient refusal of surgery.

All patients had histological confirmation except one, who was considered a high risk for biopsy due to comorbidities and low pulmonary reserve.

Pretreatment evaluation included a history and physical examination by a radiation oncologist, baseline bloodwork, and a complete staging evaluation including computed tomography (CT) of the chest and/or whole body fluorodeoxyglucose (FDG)-positron emission tomography (PET).

### Radiotherapy details

Patients were immobilized in a full-length vacuum cushion T bar immobilization. (Bionix Radiation Therapy), Patients were all scanned on GE LightSpeed RT 16 slice CT scanner. Ten respiratory phases were constructed. ITV included either maximum tumor excursion in all dimensions unless gated treatment was used. Planning was done with the ECLIPSE treatment planning system (VARIAN, CA) V13.5 release 64).

The planning target volume (PTV) equaled gross tumor volume (GTV) plus internal target volume (ITV) plus 3-4 mm. In cases of PTV and OAR overlap we prioritized OAR over PTV. The median volume of the GTV was 18 (range: 0.38–370 cm^3^). The dose to the PTV was prescribed to the 95% isodose line covering at least 95% of the PTV. Rapid arc technique was used in 26 patients and IMRT in 44 patients. All IMRT plans delivered to Arc Check (Sun Nuclear Corporation). Dose analysis performed on SNC Dose Analysis SW (Sun Nuclear Corporation). All plans had gamma passing rates for a variety of difference∕distance criteria (% by 3 mm or 2% by 2 mm) Dose-in-phantom comparisons dose-in-patient planned.

Treatment was delivered on Varian TrueBeam linear accelerators with either multiple static IMRT field or VMAT treatment. Cone beam CT was performed prior to each treatment fraction.

Initially, in this cohort patients were treated with a schedule of 8 fractions of 7.5Gy (*n* = 10), the regimen was gradually escalated to 5 fractions of 12Gy (*n* = 40), as the earlier dose was found to be tolerable. The different schedules are listed in Table 1 (BED was calculated with alpha beta ratio of 10Gy). The maximum dose constraint of the esophagus was 27 Gy, 18Gy to the spinal cord, 30 Gy to the heart, and V20</=10% to uninvolved lung tissue for the treatment naïve patients. For the reirradiated patients the only constraint that was modified was a cord maximum of 10Gy. No constraints were given to trachea or great vessels in this study.

**Table 1:** Treatment characteristics

Treatment technique (*N*)	
IMRT	44
Rapid arc	26
Biological equivalent dose, Median (range)	
< 100	5 (78-95)
> 100	65 (105-151)
Lung dose parameters	
Mean lung dose – Gy, median (range)	5.3
V5 -%, Median (range)	22.8 (1-63)
V20 -%, Median (range)	7.5 (0.1-20)
Dose to esophagus Gy (Mean)	25.8 Gy5.6-\SD+

Patients were evaluated regularly; the first follow up occurred at 6 weeks, including a PET CT and then every 3 months. Local failure was determined radiologically using PET CT.

### Statistical methods

The statistical analysis for this paper was generated using SAS Software, Version 9.4.

Continuous variables were presented by Mean ± Std, Categorical variables were presented by (N, %). Overall survival was assessed by the Kaplan Meier model with the log-rank test. Time to Failure, with death without failure as a competing risk, was assessed by the Cox proportional hazards model, with the Fine and Gray methodology. Two-sided *p* values less than 0.05 were considered statistically significant.

## Results

Seventy central lung lesions in 70 patients were treated between, February 2009 to April 2013. Ninety-eight percent of the lesions were biopsy confirmed lesions.

**Fig. 1:**
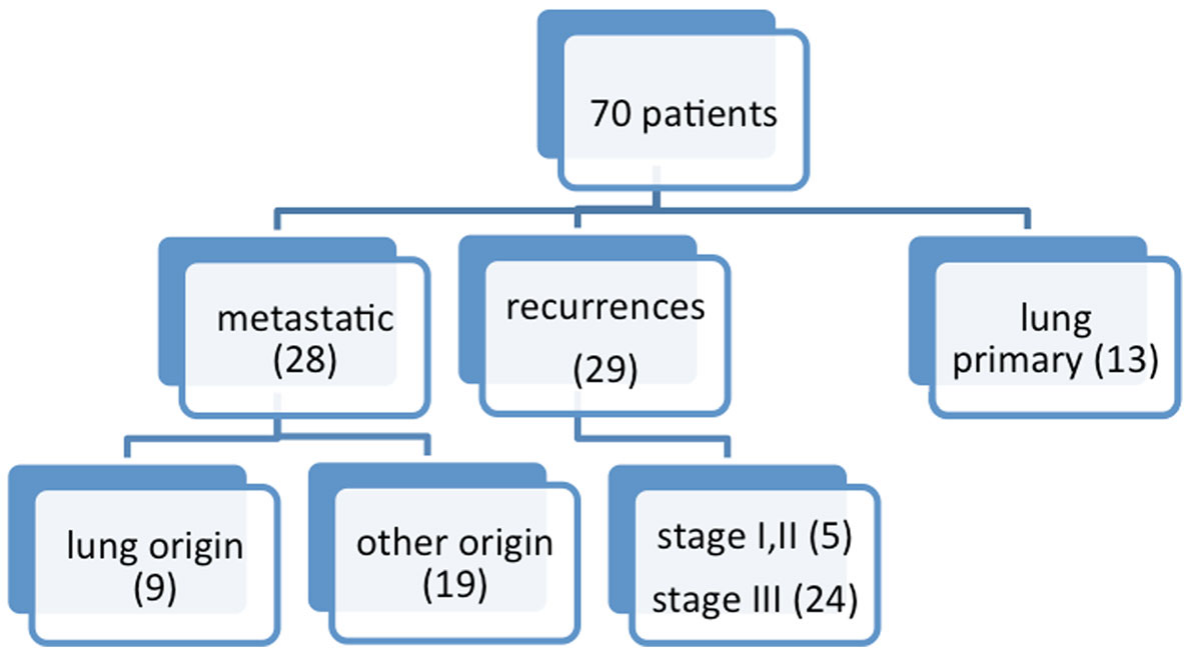
Patient distribution at recruitment

Patients included those with either primary early non-small cell lung cancer (*n* = 13) or isolated locally recurrent lung cancer (*n* = 29) and patients with pulmonary oligometastases (*n* = 28). In those patients with metastatic disease the primaries were lung [4], colorectal [5] and one thymoma, as illustrated in Fig. 1. Fifty-seven percent of the patients received BED of 132 with a schedule of 60Gy in 12 Gy per fraction, 14% received BED of 105 with total dose of 60Gy in 8 fractions. A full breakdown of dosing schedules is in Table 2.

**Table 2:** Fractionation

	BED	Number of patients
60Gy/12Gy fx.	132	40
60Gy/7.5Gy fx.	105	10
54Gy/18Gy fx.	151	6
48Gy/12Gy fx.	105	4
Other fractionation		10

56% (27) of patients had prior thoracic radiation, 51% (36) had prior surgery, 21% (15) patients had both and 31% (22) patients were treatment naïve. The patient characteristics are shown in Table 3.

**Table 3:** Patient characteristics

# of Lesions treated	70
Age (yrs) median (range)	67 (40-90)
Sex	
Male	43
Female	27
Lung primary	13
Lung recurrence	29
Stage I, II	5
Stage III	24
Metastatic lesion	28
Lung origin	9
Other origin	19
Histology	
Adenocarcinoma	22
Squamous cell carcinoma	10
Small cell lung carcinoma	6
Not specified	12
No biopsy or insufficient for DX	1
Previous radiation/surgery to lung	
Radiation	27
Surgery	36
Both	15
None	22
Gross tumor volume (cm3) Median (range)	18 (0.4-370)

### Local control

With a median follow up time of 18.3 months total of 4/70 patients experienced local failure (6%), median time to local failure was 20.4 months (CI 14-24 months). Two of the local failure patients were recurrent stage III NSCLC who were heavily pretreated with chemo-radiation and surgery.

On univariate analysis for predictors of local and regional failures, none of the variables we examined were significantly predictive of LF, including sex, age, histology, stage, previous radiation or surgery, dose of radiotherapy or GTV volume while accounting for death as a competing risk.

### Overall survival

Median OS for the whole cohort was 4.6 years (CI 3-7 years). Median OS was 7.6 years in the oligometastatic lung tumor group and was 6.4 years for patients with adenocarcinoma histology.

No other factor was found with correlation to OS specifically, sex, GTV volume, V20 and fractionation.

### Toxicity

**Fig. 2:**
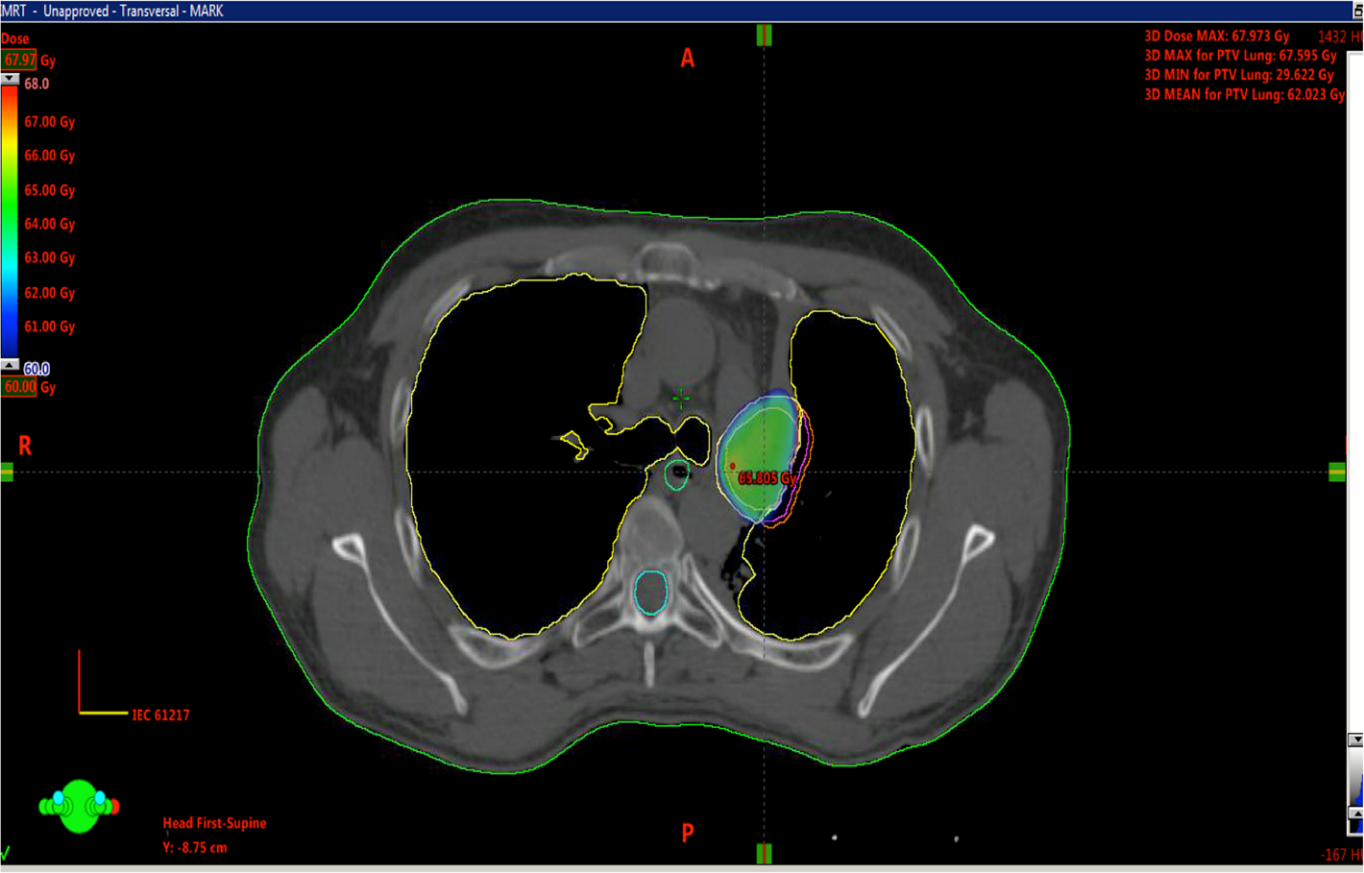
GTV volume of 38CC central tumor, treated to a BED of 132Gy

Ten patients (14%) had grade 1-2 radiation pneumonitis, there were no grade 3+ pneumonitis. One patient who had previous surgery and definitive RT developed fatal bronchial bleeding 5 months after treatment; he was treated to a BED of 132 with GTV volume of 38CC (Fig. 2).

When analyzing the patients with pneumonitis, 5/10 patients had not been previously treated. Four patients had undergone previous radiation and surgery, one patient had previous surgery alone. The mean GTV Volume was 71 cm^3^ (14-226), while mean GTV Volume for all patients was 38 cm^3^. Six patients with pneumonitis had V20 higher than 10Gy (range, 2.1-19.6Gy) and their MLD was 6.7Gy (range, 3.7-10.8Gy) but this was not statistically significant as predictors of pneumonitis.

### Patient subgroups

#### Treatment Naïve patients

Twenty-two patients who had not previously been treated (neither with radiation nor with surgery), were evaluated. Thirteen of them had early stage lung cancer, three patients were initially diagnosed with stage III lung cancer (treated with chemotherapy alone) and six patients had metastatic patients. Ninety-one percent of them received BED of 105-151 Gy and only one had a local recurrence.

#### Patients with local recurrence

Twenty-nine patients have experienced a local recurrence, 19 had previous surgery, 16 had previous radiation/chemoradiation and 8 had both. Out of the 29 patients with locally recurrent lung cancer, 24 (83%) had stage III NSCLC that recurred after initial treatment with chemo-radiotherapy [6], surgery [7] or both [4]. Only 2 of these patients had subsequent local failure with mean time to local failure of 8 months. This demonstrates the ability of SBRT to salvage patients after definitive therapy.

#### Re irradiated patients

Salvage SBRT was given to 27 patients that were previously treated with radiation to the lung and had relapsed locally, their characteristics are illustrated in Table 4. The median GTV volumes of these reiradiated patients was 18 cm^3^ (range 45–8 cm^3^).

**Table 4:** Fractionation forRe- irradiated patients

	Number of patients (27)
Fractionation (BED)	
60Gy/12Gy fx. (132)	16
60Gy/7.5Gy fx. (105)	2
54Gy/18Gy fx. (151)	3
Other fractionation (79-125)	6
Toxicity	
No toxicity	23
Grade 1 radiation pneumonitis	4
Bronchial bleeding (Fatal)	1
Local Failure	2
Median time from diagnosis to second radiation treatment	

One stage II patient who were treated upon recurrence after previous treatment with surgery and radiotherapy had local recurrence, he was treated with 36 Gy in 3 fractions to a BED of 80Gy.

#### Oligometastatic patients

Twenty-eight patients were oligometastatic patients (i.e. they presented with metastatic cancer and SBRT was offered as definitive local therapy after excellent response to systemic therapy).

These patients had central lung tumors arising from oligometastatic spread of an extrathoracic primary (19 patients) or lung cancer spread (9 patients). 22 (78%) of these patients had been treated with radiotherapy or thoracic surgery prior to SBRT, making this a heavily pretreated group. Of these patients 65% had complete response. Only 1/28 experienced local failure 17 months after treatment, again demonstrating the ability of SBRT to salvage these patients. All patients received BED above 100, 54% received a dose of 60Gy in 5 fractions.

The most frequently used regimen in this cohort was 60Gy/12 Gy per 5 fraction, BED 132 Gy, given to 57% of patients. Twelve of them were treatment naïve and all others received local treatment (radiation/chemoradiation or surgery) previously. Only 3 had subsequent local recurrence. Of note the one patient with grade 5 toxicity utilized the 12Gy*5 fractionation, however as noted previously this patient has previous chest RT to the same site.

#### Ultracentral tumors

Patients with ultracentral tumors included a total of 20 patients. All of these tumors had direct contact with the primary bronchial tree (PBT). In addition to the connection with PBT, four patients also had contact with the esophagus. The D0.5cm^3^ median was 51.9Gy and the D0.1cm^3^ was 56Gy. Of note the one patient with grade 5 toxicity as mentioned above was from this group. Excluding this patient no additional toxicity was seen in the ultracentral patient group.

## Discussion

SBRT in early stage inoperable NSCLC has been shown to achieve excellent local control and is safe. In fact, late toxicity did not appear even in long term follow up as shown in the RTOG 0236 update; at a median follow up of 4 years 55 patients were evaluable with 5 years local failure of 20%, an excess of late appearing toxicity was not observed with no treatment related death [8]. However, patients with central tumors were considered ineligible for that trial. This was due to the definition of the “No Fly Zone” in 2006 by Timmerman et al. after observing excessive toxicity with SBRT in central tumors [1].

Since then, studies have tried to ascertain the optimal regimen to treat centrally located tumors. Studies [1–3, 9] show 2-year local control of 75-95% with regimens estimated at BED_10_ of 80-120Gy, in these trials most patients did not suffer significant toxicity. Pneumonitis and bronchial stenosis were the most frequent side effects. For example, Nuyttens et al. delivered a BED of 86.4-132 Gy to 56 patients with centrally located tumors, 4 patients had grade 3 acute pneumonitis, and 6 had grade 3 late pneumonitis and 2 patients had rib fractures [9]. In 2008 Chang et al. showed LC rate of 57-100% with regimens of 40 Gyin 4 fx and 50 Gy in 4 fx in centrally located tumors less than 4 cm, the BED was 80Gy and 112.5 Gy respectively [10]. Only one patient developed brachial plexus neuropathy. In a more recent update, in 2014 100 patients were reported on with a 3 yr. LCR of 96.5% when regimens with ranging BED of 112.5 Gy and 119 Gy were given with only 2 patients having grade 3 pneumonitis [11].

However, despite these data the optimal dose and fractionation for central tumors remains a puzzle. The RTOG in trial 0813 reported in January2016 has shown that they were able to deliver 12Gy*5, with five patients suffering a DLT. They proposed the MTD at 12Gy*5 with an overall DLT of 7.2% [4]. The updated results of expanded phase II show that at the dose level 12Gy*5, 5 out of 33 patients had G3+ late toxicity with 2 yr. local control of 87.7% [12].

Given these data it appears that central SBRT is indeed safe if the fractionation is reduced to 12 Gy or below per fraction. However, BED would appear to need to be greater than 110Gy to have an effect similar to the effect as in peripheral tumors.

In our institution, we started with 8 fractions of 7.5Gy and then after initial success escalated to 60 Gy in 5 fractions. Our results show that central tumors can be treated to a BED above 100Gy with a fractionation of 60Gy in 5 fractions to a BED of 132 Gy with little toxicity and excellent local control. This dose appears to be quite tolerable especially in patients without previous RT and\or surgery to this region. This finding is similar to results recently reported by RTOG and strengthens the dose of 60Gy in five fractions as acceptable for treatment naïve patients with central disease.

**Fig. 3:**
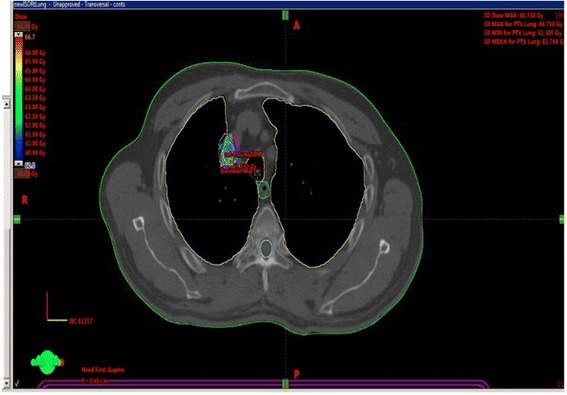
Mediastinal nodal recurrence treated with SBRT

In locally recurrent lung cancer, the data for RT is less robust. The use of conventionally fractionated radiotherapy has been reported for salvage, however, few reports regarding use of SBRT in this setting are available. At baseline, the percentage of central local recurrence following surgery for early stage lung cancer varies from report to report but is often quoted at 20% [13]. While surgical re-resection has been attempted, it is often untenable. Therefore, the option of chemoradiotherapy as salvage has been proposed. Bar et al. showed in a larger retrospective study that salvage CRT is feasible and leads to median survival of 26 months [14]. However, this approach is also fraught with significant toxicity (23% grade 4 or greater toxicity). Similar results have been published elsewhere with slightly inferior outcomes [15] Therefore, the option as we have presented of SBRT alone is quite attractive. This is especially true when the patients’ recurrence is a single station N2 node SBRT as presented may be the treatment of choice (Fig. 3).

In addition, we have shown feasibility to deliver reiradiation with SBRT following full dose definitive CRT. This is an area of significant clinical interest but is almost absent from the literature. Trovo et al. is perhaps the only similar study which showed an ability to deliver 30Gy in 5 fractions (BED 45) with 88% local control but 12% fatal complication rate [16]. Another study from the Cleveland Clinic showed 10 patients who received repeat SBRT with good results but without central radiation overlap [6]. Therefore the current study which showed in 24 patients who received previous mediastinal RT and then salvage SBRT is important when showing both safety and efficacy. Important to note, because of a single patient who after undergoing surgery and RT died after repeat SBRT we have uniformly modified our protocol to deliver only 48Gy in 4 fractions to patients who have had previous RT or Surgery and go on to receive SBRT as salvage. In addition, we are careful to limit the maximum hot spot within the central structures to 105% maximal point dose.

Finally, for patients who present with metastatic NSCLC and are able to achieve a durable remission or disease SBRT is an attractive option. Conventional radical RT has been reported by a number of groups [5, 17, 18] and clearly has been shown to be effective in metastatic patients with median PFS of 12-14 months. A prospective phase II trial demonstrated a PFS of 12 months in oligometastatic NSCLC patients treated with radical local treatment, the RT dose was 62+/−10Gy [17]. Wang et al. also showed that radical TRT (thoracic radiotherapy) with median BED of 71 Gy may have favorable outcomes for selected stage IV NSCLC patients [18].

The role of SBRT however, for oligo- metastases lie mainly in the context of commonly practiced, but rarely reported. A single trial reported by Iyengar et al. showed the ability to combine EGFR therapy with SBRT with good efficacy [19] but little has been written about other combinations. In this study we reported on two scenarios in metastatic patients for SBRT. The first, is when disease remission is achieved with chemotherapy and/or targeted therapy and SBRT is used as consolidation therapy. The second is patient that present with low volume metastatic disease in whom SBRT is part of the primary therapy. In addition to the excellent local control of SBRT reported here the overall survival is encouraging. In addition, the current study is the first to consistently utilize BED doses of above 100Gy. Our results although not directly comparable of 97% local control in these patients is quite encouraging and raises the question of what the proper dose is in these patients.

There are a number of limitations to this study. First, as in any retrospective series our patient population was heterogeneous, from the classic stage I/II primary lung tumor through the recurrent lung patient population and of the metastatic lung tumors. On the surface this makes the results less generalizable to routine stage I/II central tumors as were included in RTOG 0813. However, much evidence now exists that the radiosensitivity of tumors with SBRT is not histology dependent as a result of the method of cell death at high doses [7].

In addition, a large number of patients had previous RT and surgery to the treated area that significantly influences the potential for toxicity. In a recent review of reirradiation with SBRT after conventional radiotherapy by Amini et al. which included 7 studies to a total of 140 patients, the median thoracic radiation dose ranged from 50 to 87.5 Gy and median reirradiation doses ranged from 40 to 80 Gy LCR was 65-92%. Re irradiation was well tolerated with few grade 4-5 toxicities [20]. In our study, despite re irradiation the overall acute and late toxicity was quite minimal which has important implications for the tolerability of these doses in pretreated patients.

## Conclusions

SBRT for central tumors at was found to be safe and effective in patients with primary central disease, reiradiation, recurrence following surgery and in oligometastes. This retrospective study should prompt investigators to consider these areas for future prospective trials.

## Abbreviations

BED: Biologically equivalent dose
GTV: Gross tumor volume
LCR: Local control rate
LF: Local failure
NSCLC: Non small cell lung cancer
OS: Overall survival
PTV: Planning treatment volume
RTOG: Radiation therapy oncology group
SBRT: Stereotactic body radiotherapy
VMAT: Volumetric arc radiotherapy
